# How RAG1/2 evolved from ancestral transposases to initiate V(D)J recombination without transposition

**DOI:** 10.21203/rs.3.rs-5443361/v1

**Published:** 2025-02-12

**Authors:** Xuemin Chen, Liangrui Yao, Shanshan Ma, Xingyun Yuan, Yang Yang, Yuan Yuan, Yumei Liu, Lan Liu, Huaibin Wang, Wei Yang, Martin Gellert

**Affiliations:** Anhui university; Anhui university; Anhui university; Anhui university; Anhui university; Anhui university; Anhui university; National Institutes of Health; National Institutes of Health; NIH/NIDDK; National Institutes of Health 0000-0002-0832-4929

**Keywords:** RAG, transposition, target DNA, PHD, H3K4me3

## Abstract

The RAG1/2 recombinase, which initiates V(D)J recombination in jawed vertebrates, evolved from RNaseH-like transposases such as Transib and ProtoRAG ^1^. However, its post-cleavage transposase activity is strictly suppressed. Previous structural studies have focused only on the conserved core domains of RAG1/2, leaving the regulatory mechanisms of the non-core regions unclear. To investigate how RAG1/2 suppresses transposition and regulates DNA cleavage, we determined cryo-EM structures of nearly full-length RAG1/2 complexed with cleaved Recombination Signal Sequences (RSS) in a Signal-End Complex (SEC), at resolutions up to 2.95 Å. Two key structures, SEC-0 and SEC-PHD, reveal distinct regulatory roles of RAG2, which is absent in Transib transposase. SEC-0 displays a closed conformation, revealing that the core RAG2 facilitates sequential DNA cleavage by stabilizing the RSS-cleaved states in a “spring-loaded” mechanism. SEC-PHD reveals how RAG2’s non-core PHD and Acidic Hinge (AH) domains, which are absent in ProtoRAG, inhibit target DNA binding in transposition. Histone H3K4me3, which recruits RAG1/2 to RSS sites, does not influence RAG1/2 binding to V, D or J gene segments bordered by RSS ^2^. In contrast, the suppressed transposition can be activated by H3K4me3 peptides that dislodge the inhibitory PHD domain ^3,4^. To achieve this de-repression in vivo, however, would require an unlikely close placement of two nucleosomes flanking a target DNA bent by nearly 180°. Our structural and biochemical results elucidate how RAG1 has acquired RAG2 and utilizes its core and non-core domains to enhance V(D)J recombination and suppress transposition.

## Introduction

**Figure 1:**
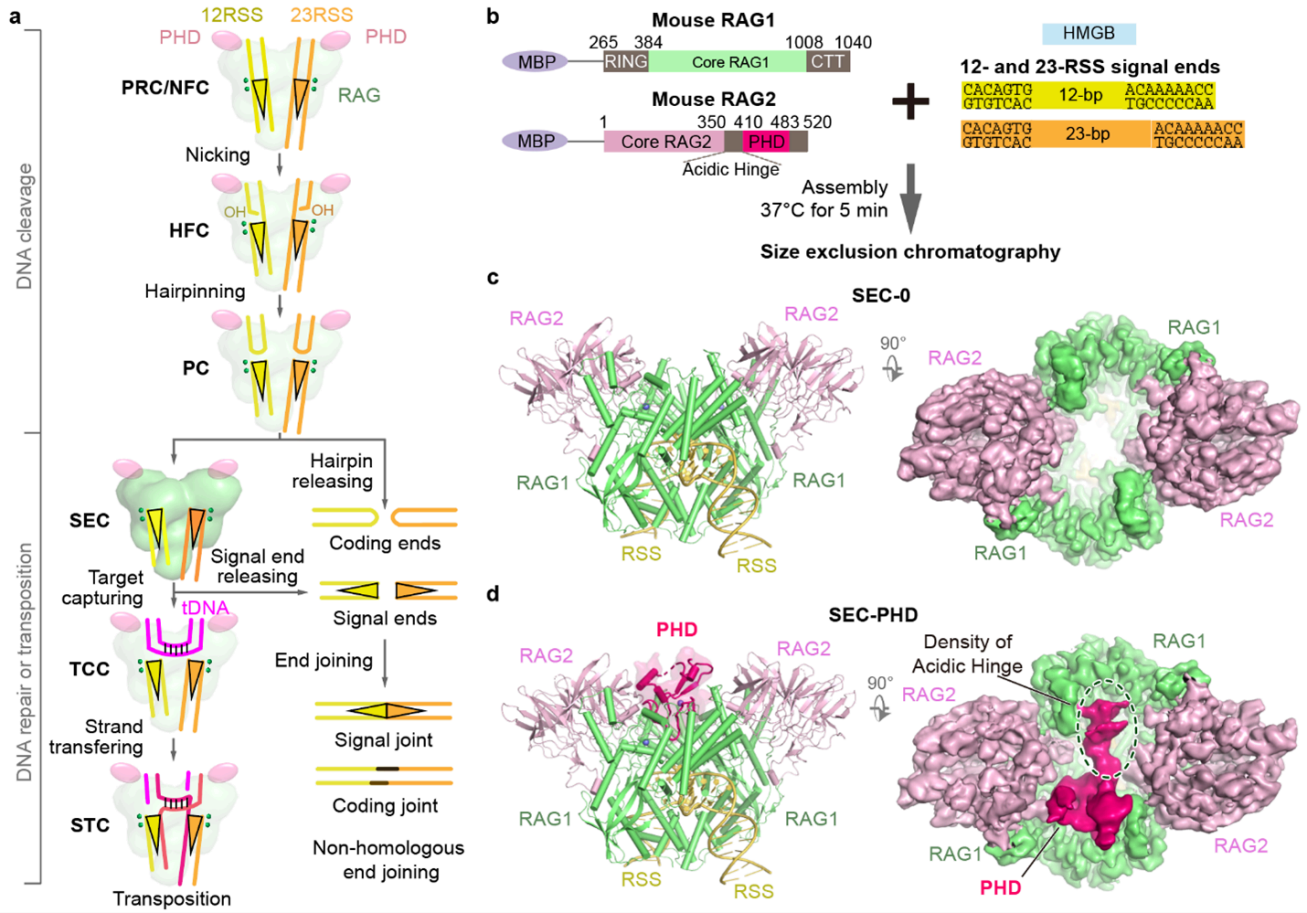
V(D)J recombination and cryo-EM structures of RAG SEC. **a**, A schematic diagram of RAG-mediated DNArecombinationand transposition. SEC is highlighted using deeper colors. **b**, Assembly of SEC for cryo-EM study and transposition assays. **c,d,** The overall structures of SEC-0 and SEC-PHD. Cryo-EM maps and models are shown as colored surface and cartoons. Map volume for the partial Acidic Hinge (AH) is marked with a black dashed ellipse.

V(D)J recombination is the essential process generating the adaptive immune system with both diversity and specificity to neutralize a great variety of infectious agents ^5^. RAG1/2 (recombination activating genes 1 and 2) protein, a heterotetramer of two RAG1 and two RAG2 subunits, cleaves at the boundaries of RSSs (recombination signal sequences) and flanking V, D and J gene segments of antigen receptors by first nicking and then hairpinning, and the resulting blunt-end signal ends and hairpin-end gene segments (also known as coding flanks) are separately re-joined by Non-Homologous End Joining (NHEJ) (Fig. 1a) ^1,5,6^. The core and non-core domains of RAG are respectively responsible for the DNA cleavage and the regulation of V(D)J recombination (Fig. 1b).

RAG1 has an RNase H-like (RNH) catalytic core domain (aa 384–1008) ^6,7^. Like all RNH-containing transposases, from bacterial Tn5, MuA, Drosophila P element, to eukaryotic Hermes and retroviral integrases ^8–11^, RAG1/2 catalytic core not only cleaves at RSS, but also can insert the two cleaved RSS ends as Terminal Inverted Repeats of transposon ends (TIR) into a new 5-bp GC-rich target site *in vitro* or *ex vivo* ^12,13^. However, the deleterious transposition by RAG1/2 is suppressed *in vivo* ^14,15^. RAG2 is absent in transposases including Transib. Beyond its core domain (aa 1–387), the regulatory PHD (aa 410–485) and Acidic Hinge (AH, aa 388–409), which are only present in jawed vertebrates and absent in RAG2L of protoRAG, have been reported to suppress transposition ^16,17^. The RAG2 PHD domain is also necessary for recruiting RAG1/2 to V, D and J gene segments on open chromatin by its binding to a histone H3K4me3 tail ^18^. Interestingly, the PHD domain also moderately autoinhibits RSS DNA cleavage, and a 21-aa H3K4me3 peptide can release this inhibition ^3,19^.

The mouse apo-RAG1/2 (mRAG) was the first core RAG structure determined ^20^. Subsequently, structures of RSS DNA binding, cleavage, and even post-cleavage transposition of RSS into a target DNA (tDNA), have been reported for mRAG, zRAG (zebra sh), Transib, and ProtoRAG ^7,17,21–27^. From DNA binding (pre-reaction complex or PRC), nick-forming (NFC) to hairpin-forming complex (HFC), RAG and related recombinases undergo an open-to-close conformational change between its two halves in the DNA cleavage process (Fig. 1a). The same closed conformation is maintained by mRAG in the strand transfer complex (STC), in which the tDNA is kinked twice by 85° (170° overall) 3 bp apart in the 5 bp 5 - CGCCG-3 target site and a further twist out of plane ^26^. The severe distortion of tDNA likely hinders RAG-mediated transposition and promotes robust disintegration to revert STC to SEC and tDNA^26,27^. The structure of the core RAG complexed with cleaved RSS signal ends, without coding flanks (SEC), has eluded characterization, and it is unclear if mRAG SEC remains closed or becomes open like Transib bound only to the cleaved TIRs ^24^.

When the PHD domain of RAG2 was included in structural studies of V(D)J recombination, a histone H3K4me3 peptide was always added to activate DNA cleavage^21,23,25–27^. As a result, PHD and the other non-core domains of RAG are disordered in all RAG structures reported to date. In the SEC mimic of zRAG in the presence of PHD and H3K4me3, the pre-cleaved RSS DNA occupies the coding flank-binding sites and resembles the HFC structure ^21^. How RAG2’s PHD inhibits RSS DNA cleavage and transposition remains unknown.

Here, we assembled mRAG SEC with the near full-length protein and the pre-cleaved RSS DNAs in the absence of any H3K4me3 peptide, determined the cryo-EM structures at up to 2.95 Å resolutions, and identified a true SEC (SEC-0) and SEC bound by PHD (SEC-PHD) (Fig. 1 and Extended Data Fig. 1). These previously unknown structures illuminate how RAG2 supports the “spring-loaded” RSS-DNA cleavage and suppresses unwanted transposition.

## Cryo-EM analysis of mRAG SEC

The mRAG SEC was assembled and puri ed using size-exclusion chromatography (see Methods). Although the elution pro le revealed a single peak. (Extended Data Fig. 2a) and SEC particles on cryo-EM grids appeared homogeneous, the volume surrounding the flank-DNA binding sites (abbreviated as flank binding site below) on 2D averages and initial 3D reconstructions was quite varied, indicating structural heterogeneity ^21^ (Extended Data Fig. 1). After local 3D classi cation of the flank binding sites, four major structural species were identified and refined. They are SEC-0, with completely empty flank binding sites (2.95 Å, C2 symmetry), SEC-1DNA, with one blunt-end RSS DNA in one of two flank binding sites (~3.4 Å), SEC-2DNA with two blunt-end DNAs in both sites (3.0 Å, C2 symmetry), and lastly SEC-PHD with a single PHD domain and AH occupying both flank binding sites (3.25 Å, C1 symmetry). The RAG structures in SEC-1DNA and SEC-2DNA are indistinguishable from mRAG HFC and zRAG bound to RSS DNAs ^21,22^. For the SEC-PHD structure, the crystal structure of PHD (PDB: 2V83) was readily docked into the large volume (~4 Å resolution) occupying the flank binding site (Fig. 1d), while AH (aa 387–409) was docked as an extended peptide into the remaining volume, but not modeled with individual residues due to the poor resolution of 6 Å or worse (Fig. 1d).

## Structure of SEC-0 and implications for recombination and transposition

**Figure 2:**
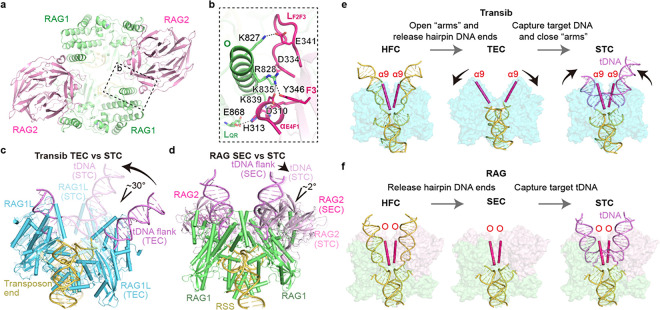
Structural comparison of Transib and RAG. **a,** Top view of SEC-0. The region shown in panel b is boxed in dashed black. **b,** The *trans* RAG1-RAG2 interactions maintaining the closed conformation in RAG SEC. **c,** Superimposition of Transib TEC and STC. tDNA flanks in Transib TEC and also in RAG SEC (d) are modeled and show as light purple cartoons. The closing motion during TEC-STC transition is indicated with black arrows. **d,** Superimposition of RAG SEC and STC. STC is expanded during SEC-STC transition and indicated with black arrows. **e,** Structural changes during Transib-mediated transpositions. The transitions from Transib HFC (PDB: 6PQX) to TEC (PDB: 6PQY) and from TEC to STC (PDB: 6PR5) show overall opening and closing motions. **f,** Structural changes during RAG-mediated transpositions. RAG HFC (PDB: 6CG0, NBD domain is omitted in the panel), SEC and STC (PDB: 6OES) overall show the conserved closed form only. Transib helix α9 (e) on ZnB domain and RAG helix O (f) on ZnH2 domain are highlighted in hotpink to show overall conformational changes.

SEC-0 as well as the other three SEC structures adopts the same closed conformation as HFC and STC, and the RAG1/2 protein chains are well superimposed among them (Cα atoms of RAG1 aa 461–1008 and RAG2 aa 1–350, RMSDs < 0.55 Å). The closed conformation of SEC-0 without mediation of the flanking DNAs in HFC or STC is maintained by the RAG1 and RAG2 interactions across the two RAG1/2 heterodimers, which we term *trans* interactions. RAG1’s helix O and loop L_QR_ interact with RAG2’s loop L_F2F3_ and helix α_E4F1_ in *trans* via extensive charge-charge interactions. For example, the K827, R828, K835 and K839 of RAG1 form salt bridges with E341, D334, and D310, and H313 of RAG2 (Fig. 2a and 2b). Even though ProtoRAG2L contains a core region similar to mRAG2, L_F2F3_ in RAG2L is much shorter than in mRAG2, and charged residues on L_F2F3_ and α_E4F1_ are not conserved. It can be expected that the trans RAG1/2L interactions are absent in ProtoRAG.

RNH-like transposases, most of which are devoid of RAG2-equivalent subunit, can bind, distort and cleave substrate dsDNA independent of a high-energy co-factor. Instead, by a hypothesized “spring-loaded” mechanism, they may use the initial protein-DNA binding energy to support the subsequent conformational changes and DNA transactions ^28^ When the apo mRAG1/2 binds to substrate RSS DNAs to form the PRC, RAG1/2 becomes very open, as if a spring is loaded, and the RAG1-RAG2 trans interaction is absent (Supplementary video 1). PRC transforms to NFC by DNA unwinding and protein domain (ZnH2) closing to nick the first DNA strand, then to the most closed HFC to cleave the second DNA strand by forming a hairpin. The SEC-0 with flanking sites empty retains the closed conformation of HFC, indicating that this closed form is the preferred and energy minimum state. Most transposases including Transib accomplish the two-step cleavage reaction without the help of RAG2, but the *trans* RAG1-RAG2 interactions and the resulting closed conformation of SEC-0 demonstrate how RAG2 supports and guides RAG1 to accomplish the “spring-loaded” mechanism in sequential DNA cleavage. While the catalytic residues reside entirely in RAG1, RAG2 becomes an essential accessary subunit to RAG1 by stabilizing RAG1 protein and enabling its “spring-loaded” mechanism of DNA cleavage ^20,29,30^.

The closed SEC renders transposition by RAG1/2 unlikely because for it to capture a target DNA, the tDNA has to either be already deformed by two 85° kinks 3 bp apart (Extended Data Fig. 4a) or wait for SEC to transiently open with all trans interactions broken. Neither scenario is of high probability. Unlike RAG1/2 recombinase, Transib lacks the RAG2 subunits, and its Transposon End Complex (TEC, equivalent to SEC) is much more open than SEC^24^ (Fig. 2c-f and Extended Data Fig. 3). To be captured by Transib, a tDNA would need to be bent by 120° instead of 170° as by mRAG. When Transib carries out transposition, from TEC to STC, it undergoes a 30° closing motion because both Transib subunits bind each flank DNA, and the cleaved tDNA is bent 150°, 30° more than the intact tDNA (Fig. 2c). For RAG1/2, SEC and STC are superimposable (Fig. 2d), and a tDNA needs to be bent 170° before transposition can take place.

## A novel PHD-AH binding site revealed by SEC-PHD

**Figure 3:**
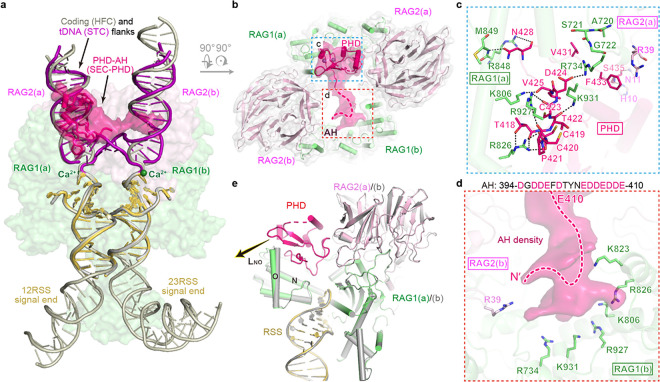
The novel PHD-AH binding site on SEC-PHD. **a,** Superimposition of SEC-PHD with HFC (PDB: 6CG0) and STC (PDB: 6OES). PHD-AH density is indicated with black arrow, and PHD is shown as hotpink cartoon with molecular surface. RSS coding and tDNA flanks are shown in purple and grey cartoon. **b,** Top view of SEC-PHD structure and the corresponding cryo-EM map. Hotpink dashed line shows the trace of AH. The regions shown in panels b and c are boxed in dashed blue and orange, respectively. **c,d** Detailed interactions of PHD and AH with the core RAG. The acidic residues in AH sequence (aa 394–410 of RAG2) are shown in hotpink letters. **e,** Structural comparison between RAG1/2 heterodimers with and without PHD binding. Colored cartoons represent the heterodimer with PHD bound and grey cartoon represents the dimer without PHD. The conformational change of helix O and LNO upon PHD binding are indicated with black arrows.

In the SEC-PHD structure, one PHD domain and the adjacent AH of RAG2 occupy the space vacated by the flank DNAs (Fig. 3a and 3b). The PHD occupies one flank binding site (called “RAG1/2 (a)”), and the AH fills the other (called “RAG1/2(b)”). These interactions are mediated by positively charged surfaces of the core RAG1/2 heterodimers and the negatively charged PHD and AH (Fig. 3c, 3d and Extended Data Fig. 2b, 2c). Loop T418-V425 of PHD interacts with the ZnH2 domain of RAG1(a). The side chains of T418 and D424 form hydrogen bonds and a salt bridge with R826 and R734, respectively (Fig. 3c). All backbone carbonyl oxygens except for D424 form polar interactions with R826, K806, R927, K931 and R734. Additionally, S435 of PHD hydrogen bonds with RAG2(a) residues H10 and N11, while N428 interacts with RAG1(a) R848 and M849. V431 interacts with RAG1(a) loop 720–722, and F433 forms a π-cation interaction with RAG2(a) R39 (Fig. 3c). Although the low-resolution map doesn’t permit a precise model of AH, aa 394–410 can be con dently located inside the positively charged tunnel lined by K823, R826, K806, R927, K931 and R734 on the RAG1/2(b), suggesting that AH imposes additional hindrance for tDNA binding (Fig. 3d).

Because the PHD domain is wider than a DNA duplex, the flank binding site is remodeled to accommodate it by opening Helix O and loop L_NO_ (aa 816–832) in the ZnH2 domain of RAG1(a) (Fig. 3e, Extended Data Fig. 4). In RAG1(a), the peptide between K817 and H818 is flipped, thus changing the conformation of loop L_NO_ and pulling it outward, which is accompanied by a 9.7° and 1.6 Å bending of a part of helix O (aa 822–832). In contrast, binding of the AH to the core RAG1 doesn’t require any conformational changes, and RAG1(b) is superimposable with SEC-0. Because one of the two PHD domains and its adjacent AH of SEC-0 occupy both flank-DNA binding sites and block tDNA from entering SEC, to eliminate the asymmetric inhibition would require eliminating binding of both PHD domains and linked AHs.

**Figure 4:**
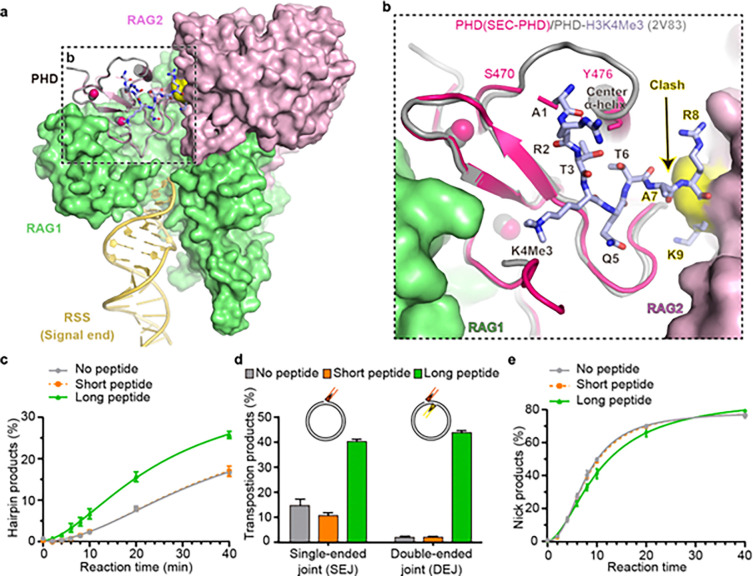
PHD-AH regulates DNA cleavage and transposition. **a,** Superimposition of PHD-H3K4me3 crystal structure (PDB: 2V83) with PHD in SEC-PHD. The region in panel b is outlined in a dashed black box. **b,** A zoom-in view of PHD domain. H3K4me3 peptide longer than 6-aa binding to PHD would clash with RAG2 and dislodge PHD from the core RAG. **c,d** Long H3K4me3 peptide (21-aa) rather than short peptide (6-aa) can dislodge PHD from RSS and tDNA binding site and stimulate RSS DNA hairpinning and transposition. Pre-nicked RSSs were used as substares in panel c. **e,** Neither long nor short H3K4me3 peptide can stimulate initial RSS DNA binding and nicking when using intact RSS substrates. The results of triplicate assays are averaged and shown.

RAG2’s PHD has been shown to bind an H3K4me3 peptide and release the autoinhibition of RSS DNA cleavage by PHD domains ^4–6^. It has been hypothesized that binding of H3K4me3 changes PHD structure and prevents PHD from associating with the core RAG1/2 ^4^. To our surprise, PHD domains in SEC-PHD and in the PHD-H3K4me3 complex (PDB: 2V83) are superimposable with an RMSD of 0.687 Å over 58 pairs of C_α_ atoms, and H3K4me3 binding appears to stabilize the otherwise flexible loop aa 471–475 in PHD domain (Fig. 4a, 4b). The buried surface of PHD in the PHD-H3K4me3 complex (735 Å^2^) is smaller than that in SEC-PHD (1,230 Å^2^), and thus H3K4me3 cannot effectively compete with SEC for PHD binding. However, we note that the H3K4me3 peptide bound to PHD in the flank-binding site would clash with the core RAG2, starting from Ala7 of histone H3 onward ^3,4^. This suggests a H3K4me3 peptide 6-residue in length would fail to release the inhibition of RSS DNA cleavage and transposition, and only longer peptides, as in nucleosomes, may dislodge and prevent the PHD from occupying the flank binding site.

Interestingly, in the apo mRAG structure, the two halves of RAG1/2 core are oriented such that even a single PHD domain cannot t (Extended Data Fig. 4e). This suggests that PHD domains cannot inhibit initial binding of V, D and J segments to RAG1/2 for RSS cleavage.

## Inucleosomes, may dislodge and prevent

To assess whether the PHD domain inhibits PRC formation (initial V, D and J substrate binding), DNA nicking, hairpinning, and transposing, and whether long (21 aa) or short (6 aa) H3K4me3 peptides can dislodge and prevent PHD from binding and inhibiting the catalytic activities of core RAG1/2, we performed DNA cleavage and transposition assays with near full-length RAG1/2, and with or without H3K4me3 peptides. Using pre-nicked or pre-cleaved RSSs to compare DNA hairpinning activity or transposition of SEC into supercoiled circular DNA target, respectively, we found that the short peptide had no effect in both reactions; but the long peptide increased the final hairpinning product by less than 2-fold and transposition product by 22-fold (double-ended joint) (Fig. 4c, 4d). First, these results confirm that our hypothesis based on the SEC-PHD structure, and the release of autoinhibition of PHD on the core RAG1/2, depends on the steric clashes between a long methylated H3 tail (such as on a nucleosome) and the RAG1/2 core region. Second, the more than 10-fold different activation levels of the long H3 peptide on hairpinning and transposition likely reflect different autoinhibition mechanisms.

V, D and J segments are connected to RSS DNA *in cis*, but a tDNA is a separate piece of DNA from RSS and thus *in trans*. Moreover, V, D and J segments do not have to bend to bind to RAG1/2 and form the PRC, unlike a tDNA to SEC. Therefore, we used uncut RSS-bordered V and J-like segments to test initial DNA binding and nicking activity. As predicted, no differences were observed among the 3 tested groups, including with or without the 21-aa H3K4me3 (Fig. 4e and Extended Data Fig. 5). In fact, the little to no alteration of initial binding and nicking in the presence of PHD and lack of stimulation by H3K4me3 peptides have been reported previously ^19,31^. In this test, we also confirm a less than 2-fold increase of hairpinning product in the presence of the long H3K4me peptide (Extended Data Fig. 5). Indeed, PHD cannot inhibit V, D or J segment binding and nicking of the first strand because binding of RSS DNA results in occupation of flank-binding sites by the coding flanks, and PHD has no chance to compete. A nicked DNA is less rigid than an intact DNA, and thus PHD free of H3K4me3 can interfere with the alignment of nicked DNA substrate for hairpin formation. But the *cis* nature of V(D)J recombination renders the inhibitory effect of PHD mild and therefore activation by H3K4me3 rather moderate compared with its effect on transposition. We deduce that acquisition of PHD serves two purposes. One is to recruit RAG1/2 to active and open chromatin domains to increase the substrate specificity of V(D)J recombination, and the second is to inhibit transposition.

## Conclusion

**Figure 5:**
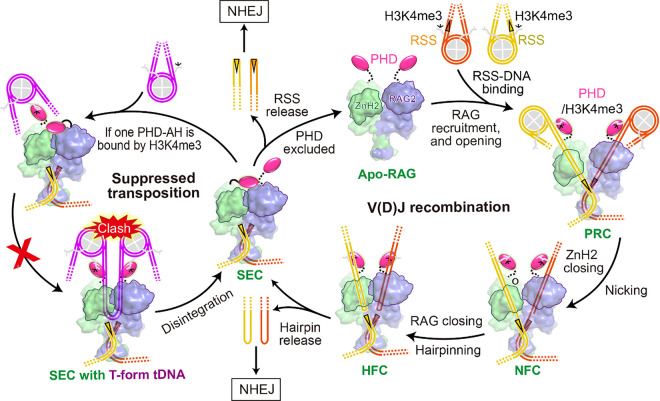
The RAG operating cycle during V(D)J recombination. Core RAG is shown in lime surface, and the core-bound and unbound PHDs are shown as solid and semi-transparent pink ovals, respectively. 12- and 23-RSSs are indicated with yellow and orange triangles. tDNA is shown with purple lines and the clash of two neighboring nucleosomes is indicated. The H3K4me3 peptide tails on nucleosomes are labeled. RAG1 ZnH_2_ domain and RAG2 (*trans*) are outlined to show the conformational changes from apo-form to HFC (SEC), and RAG stepwise becomes to the low-energy closed form. It is unlikely for SEC to capture a 170° T-form tDNA surrounded by two H3K4me3 nucleosomes. The RAG2 core and non-core domains are able to protect SEC from unwanted transposition and preserve genome stability.

With the SEC structures, we now have a complete presentation of RSS DNA cleavage and post-cleavage DNA transposition by RAG (Fig. 5). Acquisition of the core RAG2 during evolution as exemplified by ProtoRAG2L stabilizes the catalytic subunit. The later addition of the trans interaction as observed between core RAG1/2 subunits increases the “spring-loaded” DNA cleavage efficiency, and the resulting closed SEC structure presents a barrier to transposition by requiring a tDNA bent nearly 170° with further twist. The AH and PHD addition to the RAG2 core raises the anti-transposition barrier by precluding tDNA binding site in SEC, and the result is inhibition of double-end strand transfer by over 20-fold (Fig. 4). Remarkably, PHD and AH have no effect on the substrate binding and nicking steps in V(D)J recombination, and only mildly inhibit hairpin formation. RAG1/2 catalyzed processes also depend on transcription and recruitment to open chromatin via the PHD domains (Fig. 5). A pair of antigen receptor gene segments enabled by the associated nucleosomes can bind to a RAG1/2 recombinase without DNA deformation. But to dislodge two PHD domains alternately bound to an inhibited SEC, a tDNA needs to bring in two nucleosomes, one on each flanking side of the 170° bent target site, and steric clashes of such closely positioned nucleosomes and SEC may further inhibit transposition (Fig. 5). In short, the evolutionary process of RAG1/2 exemplifies how additional core and non-core domains of RAG2 eliminate unwanted transposition, while making the recombinase more specific and efficient.

## Methods

### Protein and DNA preparation

Both mouse WT RAG1 (aa 265–1040) and T490A RAG2 (aa 1–520) were N-terminally tagged by His6-MBP fusion, co-expressed in HEK293T cells and purified as previously described ^20,22^. An additional step of Mono Q anion exchange chromatography improved protein purity and removed DNA contamination. The buffer used in amylose a nity purification was 20 mM HEPES (pH 7.4), 500 mM KCl, 5% glycerol, 2 mM DTT, 0.5 mM EDTA. The salt in the protein eluate from the amylose column was diluted to 100 mM before loading onto a Mono Q column (GE Healthcare), which was pre-equilibrated with 20 mM HEPES (pH 7.4), 100 mM KCl, 5% glycerol, 2 mM DTT, 0.5 mM EDTA. mRAG was eluted by a linear gradient of 100–500 mM KCl. The puri ed mRAG was buffer-exchanged into storage buffer containing 20 mM HEPES (pH 7.4), 500 mM KCl, 20% glycerol, 0.1 mM EDTA, 2 mM DTT, concentrated to 6–8 mg/ml, and stored at −80°C. Human HMGB1 (amino acids 1–163) was prepared as reported previously ^32^. 12- and 23-RSS DNAs used for structural analyses and biochemical assays (Supplementary table 1) were synthesized as ssDNA and purified using either PAGE or HPLC method (General Biol.). Gel purified oligonucleotides were loaded onto a Glen Gel-Pak column (Glen Research) and eluted in deionized H_2_O. DNA was annealed in an annealing buffer containing 20 mM Tris-HCl, pH 8.0, 0.5 mM EDTA, 50 mM NaCl in a Thermocycler.

### Cryo-EM sample preparation and data collection

To prevent catalysis, we incubated WT mRAG, HMGB and DNAs in a Ca^2+^-containing buffer. Both RAG1 and RAG2 subunits contain a N terminal MBP-tag. MBP-mRAG protein, pre-cleaved 12- and 23-RSS signal end DNAs, and HMGB1 (aa 1–163) were mixed at 1:0.9–1.2:0.9–1.2:1.8–2.4 molar ratio in buffer containing 20 mM HEPES (pH 7.4), 100 mM KCl, 5 μM ZnCl_2_, 1 mM DTT, 5% glycerol and 5 mM CaCl_2_ and incubated at 37°C for 10 min. The mixture was further puri ed at 4°C by size exclusion chromatography on a Superdex 200 Increase 10/300 GL column (GE Healthcare) in buffer containing 20 mM HEPES (pH 7.3), 100 mM KCl, 1% glycerol, 1 mM DTT, 5 mM CaCl_2_. The elution peak fractions were pooled and used for cryo-EM grid preparation. 3 μl of the puri ed SEC (0.3 mg/ml) was spotted on freshly glow-discharged (SuPro Coolglow) QUANTIFOIL R 1.2/1.3 (Cu, 300 mesh) grids at 22°C and blotted for 5 s. The frozen grids were stored in liquid nitrogen before use.

For structure determination, the frozen grids were loaded into a Titan Krios electron microscope operated at 300 kV for automated image acquisition with SerialEM software, at the Multi-Institute Cryo-EM Facility (MICEF) of NIH. Movies were recorded on a Gatan K2 Summit direct electron detector using the super-resolution mode at 130K nominal magnification (calibrated pixel size of 1.06 Å at the sample level, corresponding to 0.53 Å in super-resolution mode) and defocus values ranging from − 0.8 to −3.0 μm. During data collection, the total dose was 70 e^−^/A^2^. The detailed collection statistics are shown in Table 1.

### Structure analysis and model refinement

Cryo-EM analysis was performed using CryoSPARC. All frames in each collected movie were aligned and summed using Patch Motion Correction, and CTF estimation were made using Patch CTF Estimation. Blob Picker and Template Picker were used for particle picking, and particles were extracted using a box size of 264 * 264 pixels. 2D classifications and 3D classifications were used to remove junk particles and select the most homogeneous particles for in-depth 3D structural analyses. The final 3D reconstruction for each class was done using Non-Uniform Refinement, and the resulting map was post-processed using DeepEMhancer^33^.

All reported resolutions are based on the “gold standard” refinement procedure and the 0.143 Fourier Shell Correlation (FSC) criterion. Local resolution was estimated using Local Resolution Estimation. For model building, STC (PDB: 6OES) and PHD (PDB: 2V83) structures were used as initial models to fit into the maps using Chimera^34^, and the resulting models were manually adjusted and rebuilt according to the cryo-EM map in COOT^35^. Phenix real-space refinement was used to re ne the models. The refinement statistics are shown in Table 1. The detailed classifications and map qualities of different conformations of SECs are shown in the Supplemental Information (Extended Data Fig. 1).

### DNA cleavage and strand transfer assays

The RSS DNA cleavage assays were performed in a reaction buffer containing 25 mM HEPES (pH 7.4), 100 mM KCl, 1 mM DTT, 0.1 mg/ml BSA, and 5 mM MgCl_2_. 200 nM each of FAM-labeled 12-RSS and 23-RSS DNAs (including coding flanks, either intact or pre-nicked, shown in Table S1) were incubated with 200 nM of heterotetrametric WT RAG, 400 nM HMGB1 and 1 μM H3K4me3 peptide (Genscript) at 37°C for 0 to 40 min. Reactions were stopped by adding an equal volume of formamide buffer (95% (v/v) formamide, 12 mM EDTA and 0.3% bromophenol blue) and heating at 95°C for 10 min. Cleavage products were separated by 19% TBE-urea PAGE, visualized and quantified using a Typhoon PhosphorImager (GE Healthcare). Plots of biochemical data show the mean ± SD from three independent experiments using Prism software.

The strand transfer (transposition) assay was carried out as previously reported^13,26^. Brie y, signal-end complex (SEC) was first assembled by mixing WT, pre-cleaved 12- and 23-RSS signal ends without coding flank and HMGB1 at 1:1:1:2 molar ration in a pre-reaction buffer (25 mM HEPES (pH 7.4), 100 mM KCl, 1 mM DTT, and 0.2 mM CaCl_2_) at 37°C for 10 min. The strand transfer rection was carried out by mixing 200 ng supercoiled pUC19 plasmid, 300 nM SEC with 20 μM H3K4me3 peptide in a reaction buffer (25 mM HEPES (pH 7.4), 100 mM KCl, 1 mM DTT, 0.1mg/ml BSA and 5 mM MgCl_2_) and incubating at 37°C for 1 h. The reaction was stopped by adding 25 mM EDTA, and proteins were removed by incubation with 0.4 mg/ml Proteinase K for 30 min at 37°C. DNA products were resuspended in 15 ul loading buffer after ethanol precipitation and separated on a 1.5% agarose gel by electrophoresis. DNA bands were stained with ethidium bromide and quantified using a Typhoon Phosphorimager (GE Healthcare). Data from three independent experiments are averaged and shown with standard deviations using Prism software.

## Figures and Tables

**Table 1.** Statistics of cryo-EM data collection and structure refinement

	SEC-0 (EMDB: EMD-61717) (PDB: 9JPX)	SEC-PHD (EMDB: EMD-61715) (PDB: 9JPU)	SEC-1DNA (12RSS side) (EMDB: EMD-61816) (PDB: 9JTS)	SEC-1DNA (23RSS side) (EMDB: EMD-61817) (PDB: 9JTU)	SEC-2DNA (EMDB: EMD-61730) (PDB: 9JQN)

**Data collection and processing**					
Magnification	130,000	130,000	130,000	130,000	130,000

Voltage (kV)	300	300	300	300	300

Electron exposure (e^−^/Å^2^)	60	60	60	60	60

Defocus range (μm)	−0.8 to −3.0	−0.8 to −3.0	−0.8 to −3.0	−0.8 to −3.0	−0.8 to −3.0

Pixel size (Å)	1.06	1.06	1.06	1.06	1.06

Symmetry imposed	C2	C1	C1	C1	C2

Initial particle images (no.)	6,917,387	6,917,387	6,917,387	6,917,387	6,917,387

Final particle images (no.)	260,975	57,418	90,752	72,732	144,322

Map resolution (Å) (FSC threshold=0.143)	2.95	3.25	3.36	3.43	3.03

**Refinement**					

Initial model used (PDB code)	9JPU	6OES	9JPU	9JPU	9JPU

Model resolution (Å) (FSC threshold=0.5)	3.2	3.6	3.7	3.7	3.3

Model composition					
Non-hydrogen atoms	15,399	15,963	18,710	18,710	16,488
Protein residues	1,784	1,853	1,928	1,928	1,784
Ligands (nucleotide)	58	58	164	164	112

*B* factors (Å^2^)					
Protein	64.77	77.87	73.18	72.61	56.11
Ligand (nucleotide)	77.33	96.33	152.32	148.34	93.12

R.m.s. deviations					
Bond lengths (Å)	0.539	0.471	0.579	0.575	0.587
Bond angles (°)	0.006	0.002	0.003	0.003	0.003

Validation					
MolProbity score	1.94	1.78	1.81	1.85	1.78
Clashscore	6.12	6.39	10.24	10.41	9.07
Rotamers outliers (%)	2.29	1.84	0.18	0.12	0.26

Ramachandran plot					
Favored (%)	95.32	96.51	95.92	95.45	95.71
Allowed (%)	4.68	3.43	4.08	4.55	4.12
Disallowed (%)	0	0.05	0	0	0.17

## Data Availability

The accession numbers for the cryo-EM structures and associated density maps of the mouse SEC-0, SEC-PHD, SEC-12DNA, SEC-23DNA, SEC-12/23DNA complexes reported in this paper have been deposited to the PDB and EMDB under accession PDB codes 9JPX, 9JPU, 9JTS, 9JTU and 9JQN, and EMD-61717, EMD-61715, EMD-61816 EMD-61817 and EMD-61730, as specified in Table 1.
